# Changes in the cellular immune system and circulating inflammatory markers of stroke patients

**DOI:** 10.18632/oncotarget.12201

**Published:** 2016-09-22

**Authors:** Chao Jiang, Weixia Kong, Yuejuan Wang, Wendy Ziai, Qingwu Yang, Fangfang Zuo, Fangfang Li, Yali Wang, Hongwei Xu, Qian Li, Jie Yang, Hong Lu, Jiewen Zhang, Jian Wang

**Affiliations:** ^1^ Department of Neurology, The Fifth Affiliated Hospital of Zhengzhou University, Zhengzhou, Henan, China; ^2^ Department of Anesthesiology/Critical Care Medicine, Johns Hopkins University, School of Medicine, Baltimore, MD, USA; ^3^ Department of Ultrasonography, The Fifth Affiliated Hospital of Zhengzhou University, Zhengzhou, Henan, China; ^4^ Department of Neurology, Johns Hopkins University, School of Medicine, Baltimore, MD, USA; ^5^ Department of Neurology, Xinqiao Hospital, Third Military Medical University, Chongqing, China; ^6^ Department of Radiology, The Fifth Affiliated Hospital of Zhengzhou University, Zhengzhou, Henan, China; ^7^ Department of Neurology, The First Affiliated Hospital of Zhengzhou University, Zhengzhou, Henan, China; ^8^ Department of Neurology, Peoples Hospital of Zhengzhou University, Zhengzhou, Henan, China

**Keywords:** ischemic stroke, lymphocyte subpopulations, circulating inflammatory markers, infection, immunosuppression

## Abstract

This study was designed to investigate dynamic changes in the cellular immune system and circulating inflammatory markers after ischemic stroke. Blood was collected from 96 patients and 99 age-matched control subjects for detection of lymphocyte subpopulations and inflammatory markers. We observed decreases in B cells, Th cells, cytotoxic T cells, and NK cells and an increase in regulatory T (Treg) cells in stroke patients on days 1, 3, and 7. Serum levels of TNF-a, C-reactive protein (CRP), IL-4, IL-6, IL-10, IL-17, IL-23, and TGF-β increased, whereas serum level of IFN-? decreased at all time points after stroke. Stroke patients with infection exhibited a similar tendency toward changes in some lymphocyte subpopulations and inflammatory markers as stroke patients without infection. After controlling for NIH Stroke Scale (NIHSS), we observed no differences in lymphocyte subpopulations between patients with anterior circulation stroke and those with posterior circulation stroke at any time point. The splenic volume correlated positively with the percentages of B cells, Th cells, and cytotoxic T cells, but negatively with Treg cells on day 3 after stroke. Infections were associated with splenic volume, leukocyte counts, percentage of Treg cells, and serum levels of CRP, IL-10, and IFN-? on day 3. Lesion volume correlated positively with CRP, IL-6, and IL-23, but negatively with IFN-? on day 3. The NIHSS showed a positive relation with IL-6 and IL-10 on day 3. Ischemic stroke has a profound effect on the systemic immune system that might explain the increased susceptibility of stroke patients to infection.

## INTRODUCTION

Studies have shown that the probability of infection in patients with stroke is 21-65% [1, 2]. Although clinical studies suggest that reduction of bulbar reflexes, drowsiness, dysphagia, and subsequent aspiration strongly increase the incidence of infections [1, 3, 4], recent preclinical evidence suggests that stroke-induced immunosuppression may also play a decisive role in infection risk [1, 5].

Stroke-induced immunodepression syndrome (SIDS) may involve interaction between the cellular immune system and circulating inflammatory markers [6–8]. Some research has shown that regulatory T (Treg) cells, interleukin (IL)-4, IL-10, and transforming growth factor (TGF)-β contribute to the process of immunodepression [9–11]. Moreover, TGF-β and IL-10 are essential for the differentiation of Treg cells [12]. On the other hand, IL-17 and IL-23 are critical for the differentiation of some types of helper T cells (such as Th17) and contribute to the inflammatory reaction process [12–14]. Several studies have shown that the percentages of cytotoxic T cells, natural killer (NK) cells, and B lymphocytes, and serum levels of TNF-α, IFN-γ, C-reactive protein (CRP), IL-4, IL-6, IL-10, and TGF-β are altered after stroke [15–17]. However, few studies have examined the dynamic changes in percentages of Treg cells and Th cells, or in the levels of IL-17 and IL-23 after stroke. No studies have investigated differences in the cellular immune system and circulating inflammatory markers between stroke patients with and without infection, or assessed the influence of vascular territories on the severity of SIDS. Furthermore, little is known about the correlation between splenic volume and lymphocyte subpopulations, or the relationship between lymphocyte subpopulations, cytokine levels, and infections in patients with ischemic stroke.

Therefore, we evaluated changes in the frequency or percentage of peripheral blood lymphocyte subpopulations and levels of inflammatory markers in blood samples of patients during the first 7 days after ischemic stroke. We also measured spleen size and rates of pneumonia and urinary tract infection. Then we ascertained the correlation between splenic volume and lymphocyte subpopulations, and the influence of vascular territories on the severity of SIDS. We also evaluated the relationship between lymphocyte subpopulations, cytokine levels, and infections and determined whether changes in circulating inflammatory markers are associated with stroke lesion volume and/or neurologic function in patients with cerebral infarction.

## RESULTS

### Baseline characteristics of the two groups

The stroke group comprised 59 men and 37 women with an average age of 58.9 years (range, 22-91). The control group was composed of 56 men and 43 women with an average age of 60.8 years (range, 20-86). Based on the Trial of Org 10172 in Acute Stroke Treatment (TOAST) classification [18], strokes were attributed to large-artery atherosclerosis (17), cardioembolic stroke (33), small-artery occlusion or lacunes (11), other determined cause (3), and undetermined cause (32). The differences in demographic variables and comorbidities between the two groups are shown in Tables 1 and 2 [19]. Three patients with stroke died within 3 days of onset and five died within 7 days.

**Table 1:** Characteristics of control subjects and stroke patients

Characteristic	Control group (n=99)	Stroke group (n=96)	Statistical quantity	*p*
Age (mean ± SD)	60.79±18.30	58.93±16.44	t = 0.744	0.458
Sex (male)	56	59	χ2 = 0.032	0.858
Hypertension	27	29	χ2 = 0.032	0.858
Diabetes	21	26	χ2 = 0.511	0.475
Dyslipidemia	31	30	χ2 = 0.129	0.720
Obesity (BMI ≥ 30) (%)	14.1	17.7	χ2 = 0.464	0.496
Smoking (%)	21.2	22.9	χ2 = 0.082	0.774
AB pointsa			t = 5.850	0.119
0	48	32		
1	39	43		
2	9	15		
3	3	6		
Atrial fibrillation	5	7	χ2 = 0.424	0.362
LVEF	64.50±7.58	62.70±7.79	t =1.640	0.103
Posterior circulation stroke	N/A	29	N/A	N/A
Time since stroke (mean ± SD)	N/A	(7.65±2.51) h	N/A	N/A
NIHSS score, median (range)	N/A	9 (7-13)	N/A	N/A
Lesion volume, cm3 (mean ± SD)	N/A	23.89±9.49	N/A	N/A

AB = atherosclerotic burden; BMI = body mass index; LVEF = left ventricular ejection fraction; N/A = not applicable.

^a^ AB was evaluated with the following scores: 0 points, <3 vascular risk factors and no effect on other vascular territories (coronary artery disease or peripheral artery disease); 1 point, ≥3 risk factors; 2 points, 1 vascular territory affected (in addition to cerebrovascular); and 3 points, 2 vascular territories affected. AB scores of 2 and 3 were independent of the number of vascular risk factors.

**Table 2:** Characteristics of stroke patients with or without infection

Characteristic	Infection (n= 51)	No infection (n= 45)	Statistical quantity	*p*
Age (mean ± SD)	59.06±14.40	58.79±18.66	t = 0.078	0.938
Sex (male)	27	22	χ2 = 0.157	0.692
Hypertension	16	13	χ2 = 0.070	0.791
Diabetes	15	11	χ2 = 0.299	0.585
Dyslipidemia	17	13	χ2 = 0.220	0.639
Obesity (BMI ≥ 30) (%)	19.6	15.6	χ2 = 0.269	0.604
Smoking (%)	25.5	20	χ2 = 0.408	0.523
AB pointsa			t = 1.426	0.699
0	19	13		
1	25	18		
2	9	6		
3	5	1		
Atrial fibrillation	5	2	χ2 = 1.016	0.314
LVEF	62.10±8.31	63.22±7.34	t = 0.701	0.485
Posterior circulation stroke	20	9	χ2 = 4.187	0.041
NIHSS score, median (range)	9 (7-15)	8 (6-10)	t = 3.205	0.002
Lesion volume, cm3 (mean ± SD)	27.20±9.90	20.15±7.50	t = 3.888	<0.001

AB = atherosclerotic burden; BMI = body mass index; LVEF = left ventricular ejection fraction.

^a^ AB was evaluated with the following scores: 0 points, <3 vascular risk factors and no effect on other vascular territories (coronary artery disease or peripheral artery disease); 1 point, ≥3 risk factors; 2 points, 1 vascular territory affected (in addition to cerebrovascular); and 3 points, 2 vascular territories affected. AB scores of 2 and 3 were independent of the number of vascular risk factors.

### Spleen size and incidence of infection

**Figure 1:**
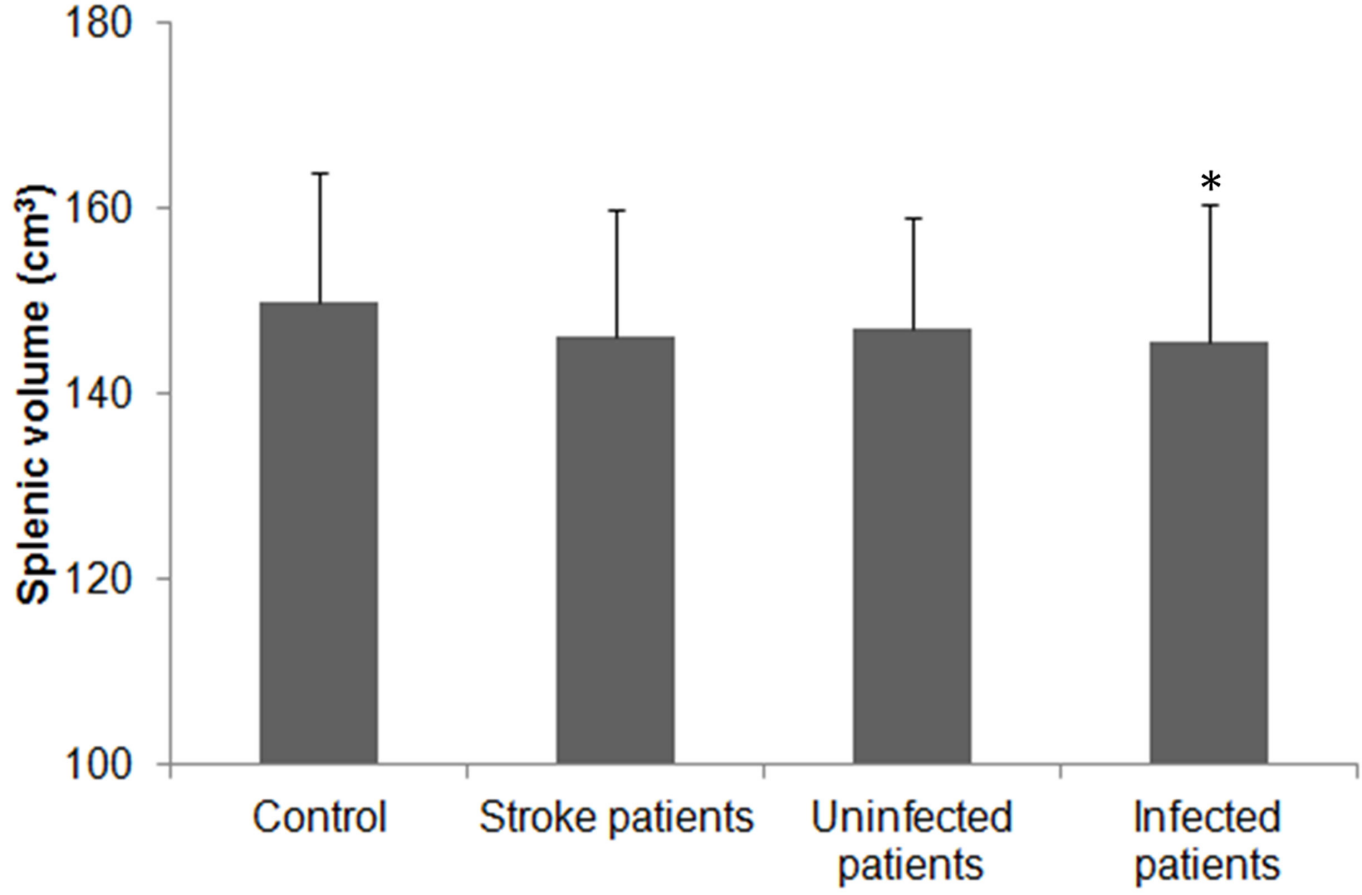
Splenic volume in patients with ischemic stroke Splenic volume of control subjects and patients with ischemic stroke (total, with and without infectious complications). The splenic volumes of patients with ischemic stroke were slightly reduced but did not differ significantly from that of the control group (p > 0.05). Splenic volume did not differ significantly between uninfected stroke patients and control subjects (p > 0.05), but it was significantly lower in patients with infection than in control subjects (*p < 0.05).

The mean splenic volume was not significantly smaller in the stroke group (146.25±13.71 cm3) than in the control group (150.05±27.71 cm3, p = 0.055), but it was significantly smaller in stroke patients with infection (n = 51; 145.54±15.10) than in control subjects (p = 0.041). No significant difference was observed between infected patients and uninfected patients (147.05±12.01 cm3), or between uninfected patients and control subjects (p = 0.591 and 0.146, respectively; Figure 1). Infections occurred in 51 (53.1%) of 96 ischemic stroke patients during the first week of hospitalization (5.1±2.8 days); 36 (37.5%) developed pneumonia, and 20 (20.8%) developed a urinary tract infection. Among the patients with infections, 11 (11.5%) developed sepsis. All patients diagnosed with a bacterial infection received antimicrobial agents according to their symptoms and signs, routine blood and urine laboratory test results, and pathogenic examination results.

### Lymphocyte subpopulations

**Figure 2:**
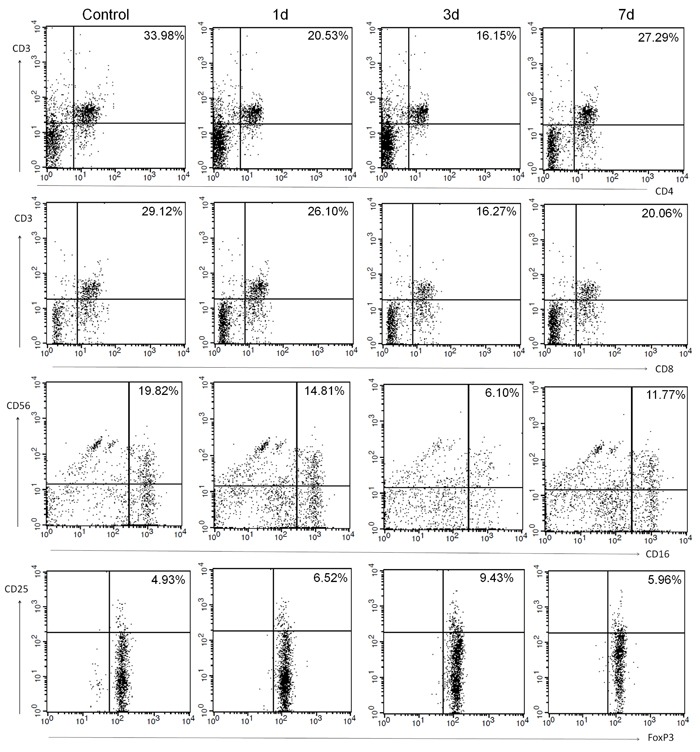
Representative flow cytometric analyses of blood lymphocyte subpopulations in control subjects and in patients with ischemic stroke on days 1, 3 and 7: Th cells (CD3 +CD4+), cytotoxic T cells (CD3+CD8+), NK cells (CD16+CD56+), and Treg cells (CD4+CD25+FoxP3+).

**Figure 3:**
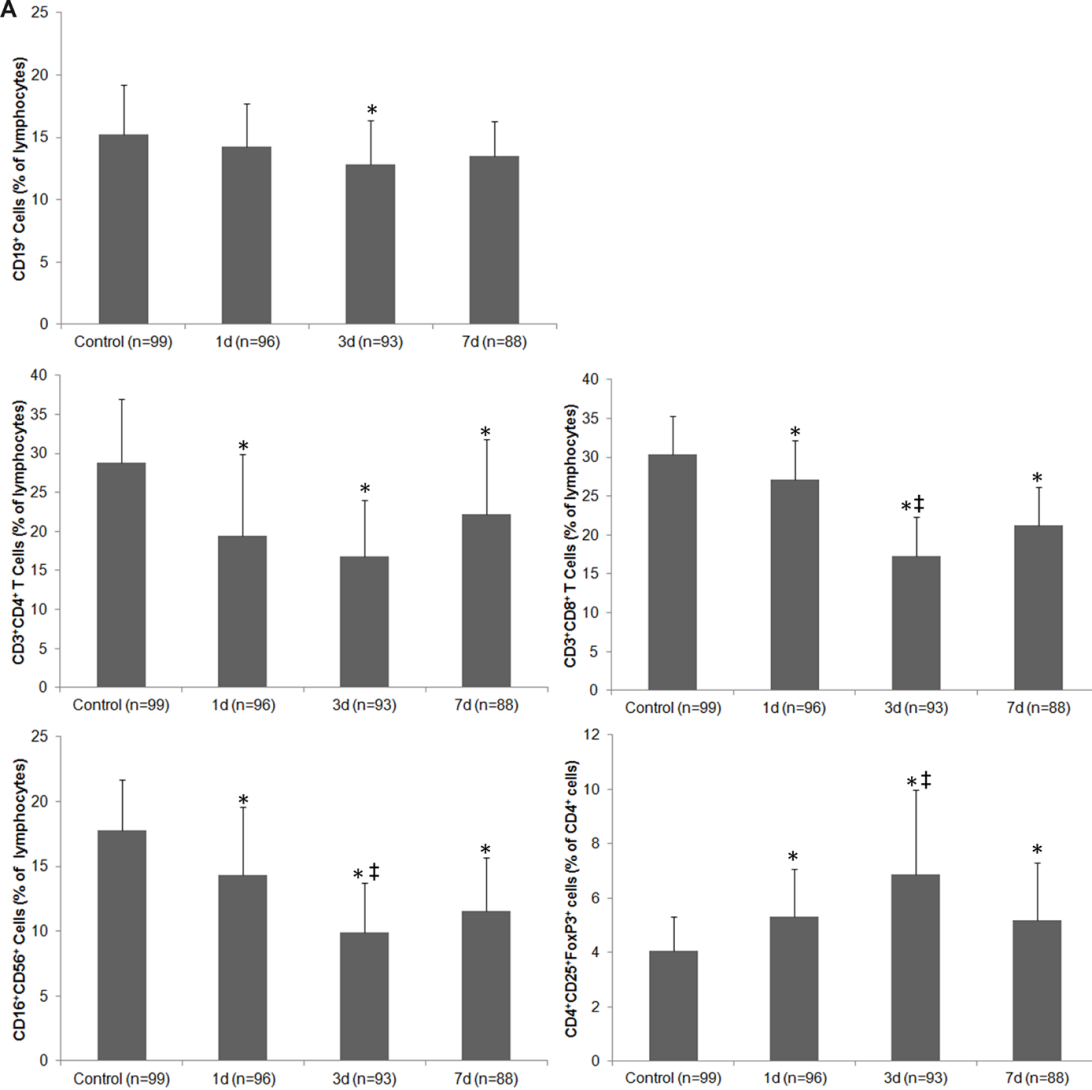

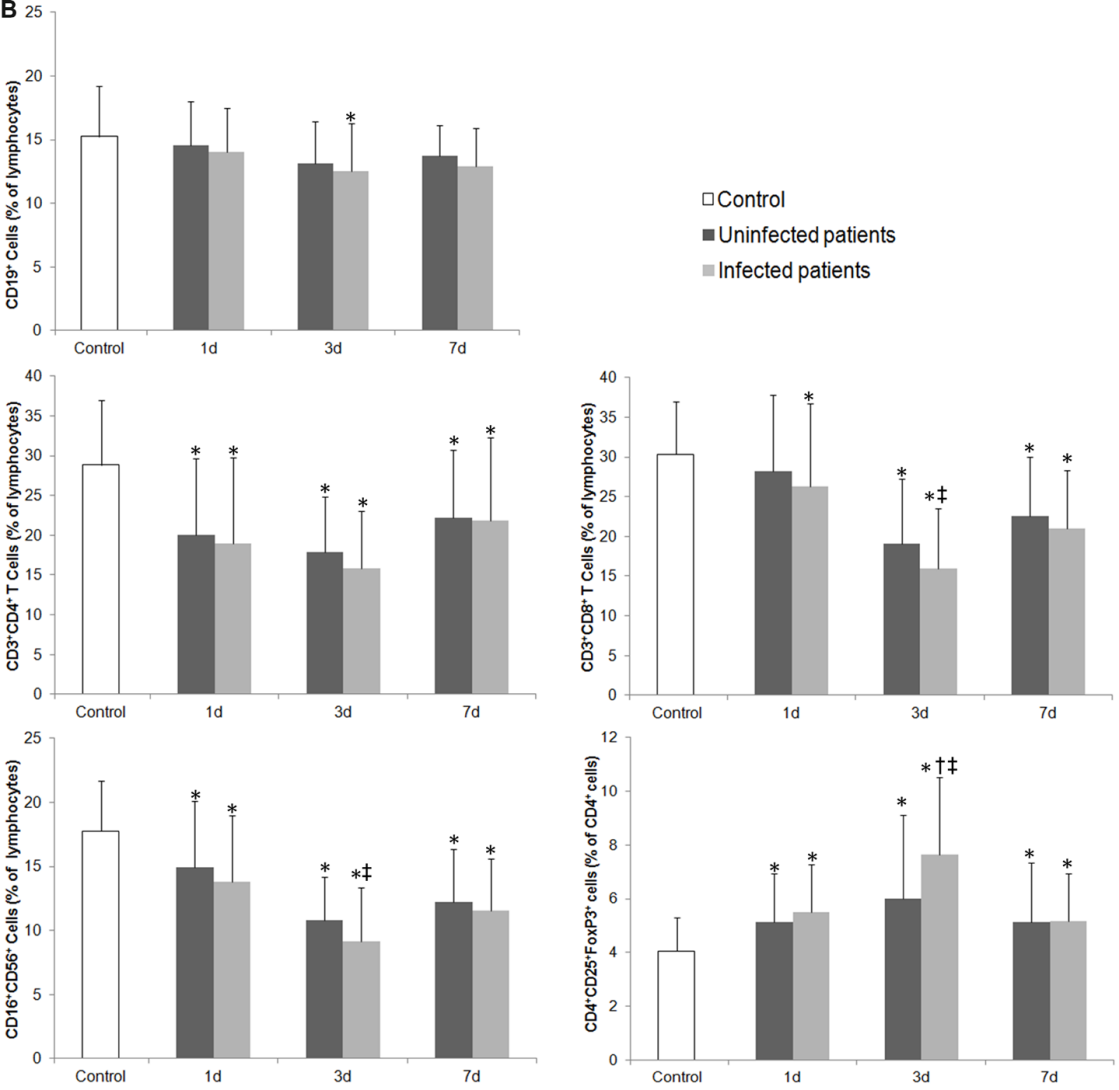

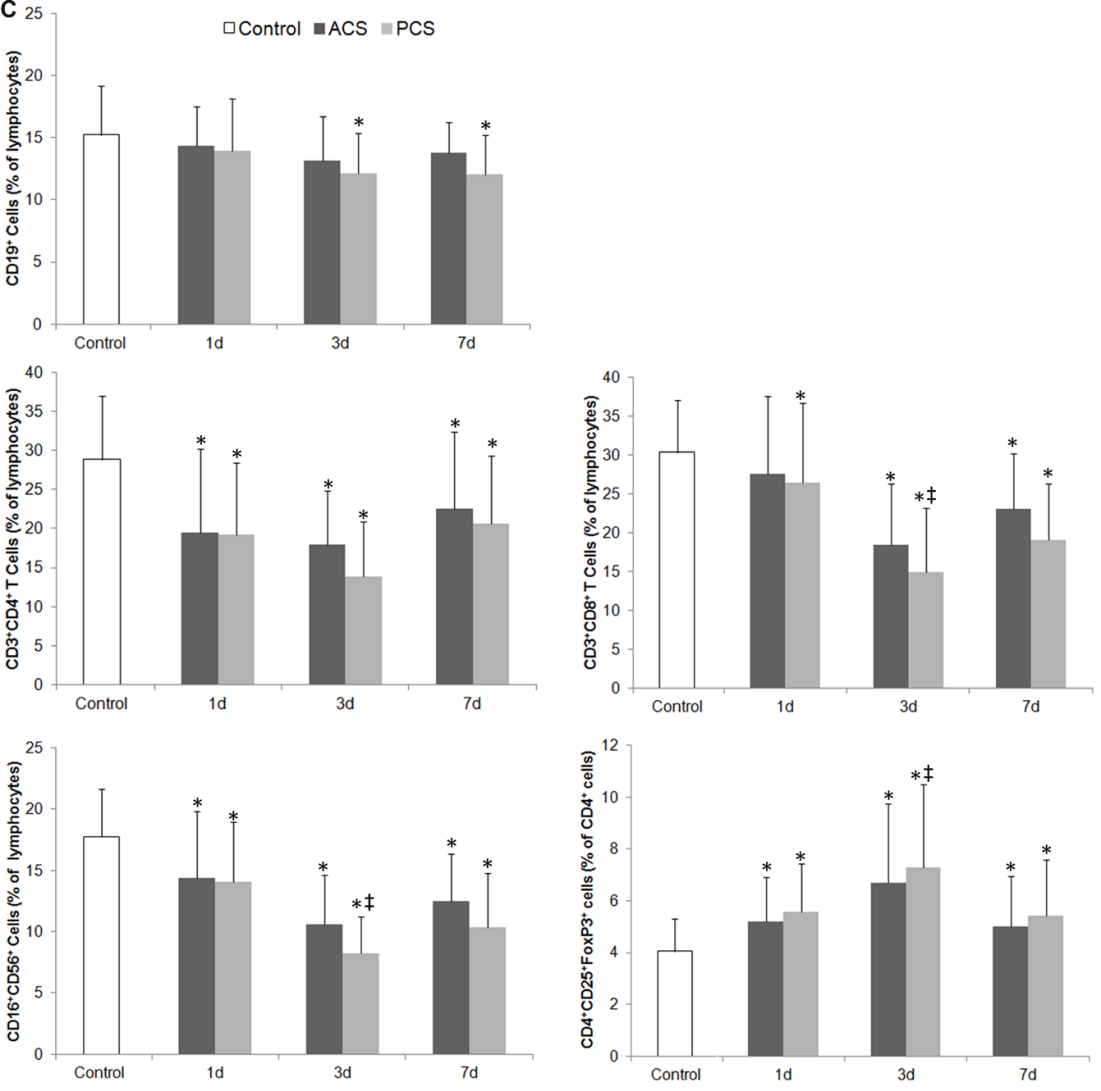
Changes in the cellular immune system of patients with ischemic stroke **A.** The percentage of lymphocyte subpopulations in control subjects and in stroke patients on days 1, 3 and 7. **B.** The percentage of lymphocyte subpopulations in control subjects and in stroke patients with and without infectious complications on days 1, 3 and 7. **C.** The percentage of lymphocyte subpopulations in control subjects and in patients with anterior circulation strokes (ACS) and posterior circulation strokes (PCS) on days 1, 3 and 7 after controlling for NIHSS. * p < 0.05 vs. controls; † p < 0.05 vs. stroke patients without infections; ‡ vs. day 1 after stroke. Data represent mean ± SD.

The temperature of the control group was 36.2°C (SD = 0.45) on the date of blood collection. The temperature of the stroke group was 37.2°C (SD = 0.61) on day 1, 37.7°C (SD = 0.94) on day 3, and 36.8°C (SD = 0.73) on day 7 (F = 102.77; p < 0.01 vs. control on all days). The median number of leukocytes in the control group was 5.23×109/L, whereas the median in the stroke group was 11.96×109/L, 9.37×109/L, and 10.41×109/L on days 1, 3, and 7, respectively (F = 70.99; p < 0.01 vs. control on all days). Representative dot plots of flow cytometric analyses in Figure 2 show blood lymphocyte subpopulations in stroke patients and control subjects. The percentages of Th cells, cytotoxic T cells, and NK cells were lower in the stroke group than in the control group on days 1, 3, and 7 (all p < 0.05), but the percentage of B lymphocytes was lower than control only on day 3 (p < 0.05); the percentage of Treg cells was increased in stroke patients on all three days when compared with that in the control group (all p < 0.01; Figure 3A). When patients were stratified into infected and uninfected groups, we observed that the percentage of B lymphocytes was lower in infected patients than in controls on day 3. Notably, we found similar changes in lymphocyte subpopulation percentages in infected and uninfected groups at most of the three time points when compared with populations in the control subjects (Figure 3B). However, only the percentage of Treg cells was higher in infected patients than in uninfected patients on day 3 after stroke onset (p < 0.05), and no differences were found in the percentages of B cells, Th cells, cytotoxic T cells, and NK cells between infected patients and uninfected patients at any time points (all p > 0.05; Figure 3B). When patients were stratified into anterior circulation stroke (ACS) and posterior circulation stroke (PCS), we observed that the percentage of B lymphocytes was lower in patients with PCS than in controls on day 3; percentages of Th cells, cytotoxic T cells, and NK cells were lower and percentage of Treg cells were higher in patients with ACS and PCS than in control subjects at most of the three time points (Figure 3C). Further analysis revealed no significant differences in lymphocyte subpopulations between patients with ACS and PCS at any time point after controlling for NIHSS (all p > 0.05; Figure 3C).

### Serum level of circulating inflammatory markers and cytokines

**Figure 4:**
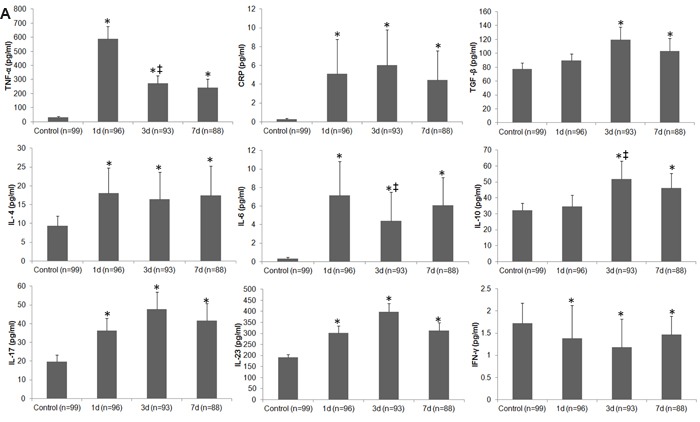

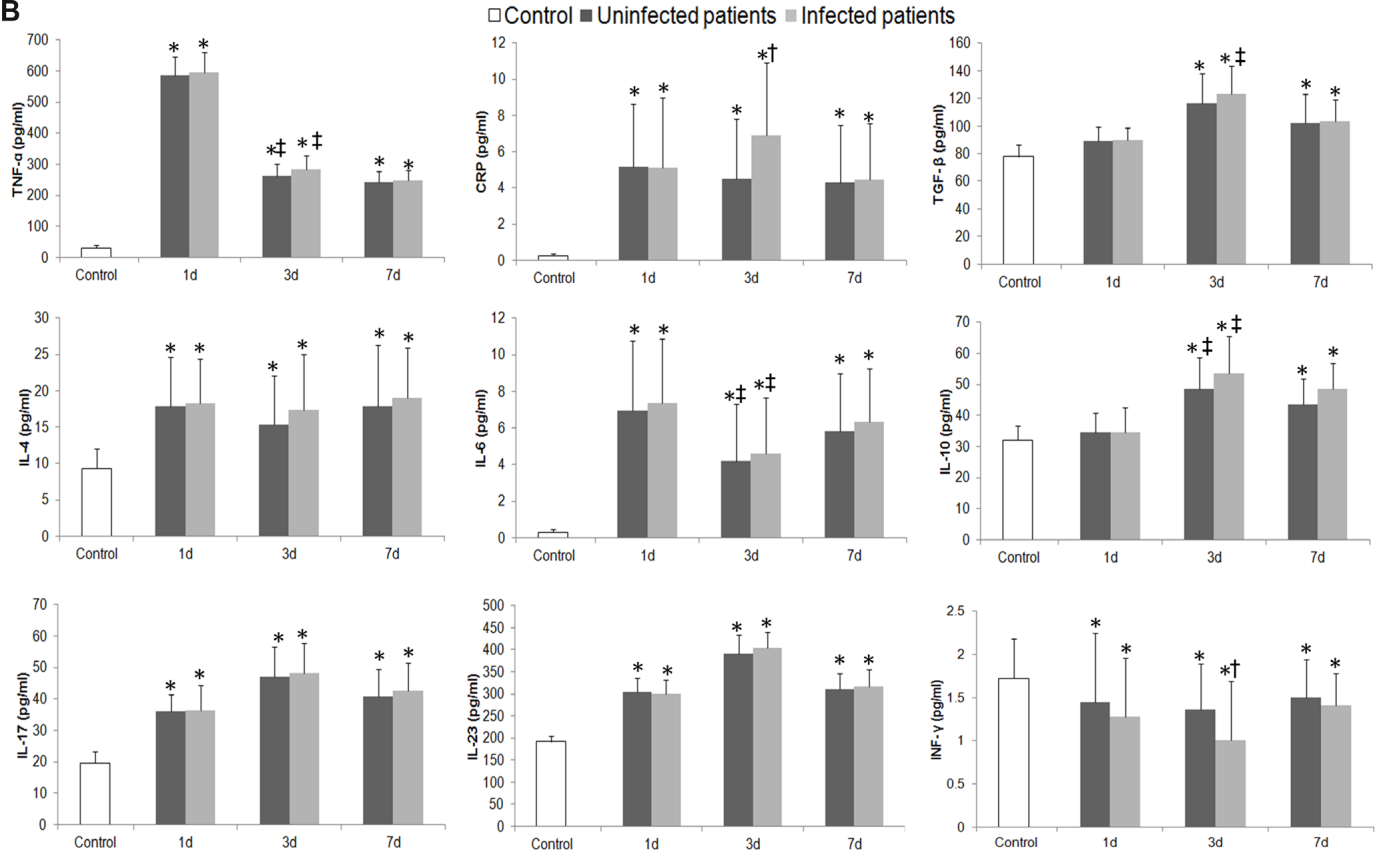
Differences in circulating inflammatory markers between control subjects and patients with ischemic stroke **A.** Changes in serum levels of inflammatory markers in control subjects and in stroke patients on days 1, 3, and 7. **B.** Changes in serum levels of inflammatory markers in control subjects and in stroke patients with and without infectious complications on days 1, 3 and 7. *p < 0.05 vs. controls; †p < 0.05 vs. stroke patients without infections; ‡ vs. day 1 after stroke. Data represent mean ± SD.

The serum levels of TNF-α, CRP, TGF-β, IL-4, IL-6, IL-10, IL-17, and IL-23 were all increased on days 1, 3, and 7 after stroke when compared with levels in the control group (all p < 0.05; Figure 4A); however, the content of IFN-γ was lower in stroke patients than in controls at each time point (p < 0.01; Figure 4A). Moreover, serum levels of TNF-α and IL-6 decreased, whereas IL-10 increased in stroke patients between day 1 and day 3 (all p < 0.05, Figure 4A). When patients were stratified by infection status, we observed increases in TNF-α, CRP, TGF-β, IL-4, IL-6, IL-10, IL-17, and IL-23, and a decrease in IFN-γ in both uninfected and infected patients when compared with control subjects at most of the three time points (Figure 4B). We also compared infected and uninfected patients at each time point. Serum CRP level was higher in infected patients than in uninfected patients, but IFN-γ level was lower in the infected patients on day 3 after stroke onset (both p < 0.05). Serum levels of IL-10 in infected patients were not different from those in uninfected patients on days 3 and 7 after stroke onset (p > 0.05; Figure 4B).

### Correlation between spleen size and lymphocyte subpopulations in stroke patients

On day 3 after stroke onset, we found an independently positive correlation between splenic volume and the percentages of circulating B cells, Th cells, and cytotoxic T cells (all p < 0.05; Table 3). However, the percentage of circulating Treg cells was negatively correlated with splenic volume (p < 0.05; Table 3).

**Table 3:** Correlation coefficients between spleen size and lymphocyte subpopulations on day 3 in stroke patients

Parameter	B cells	Th cells	Cytotoxic T cells	NK cells	Treg cells
*r*	0.347	0.228	0.223	0.091	-0.264
*p*	0.001*	0.023*	0.026*	0.378	0.008*

* Statistically significant.

NK = natural killer; Th = helper T; Treg = regulatory T.

### Relationship between cytokine levels, lymphocyte subpopulations, and infections

Adjusted logistic regression analysis revealed that infection was independently associated with splenic volume, leukocyte counts, the percentage of Treg cells, and the serum levels of CRP, IL-10, and IFN-γ on day 3 after stroke (all p < 0.05; Table 4).

**Table 4:** Multivariate analysis: the relationship between cytokine levels, lymphocyte subpopulations, and infections in stroke patients

Parameter (day 3)	OR	95% CI	*p*
Splenic volume	0.120	0.019–0.799	0.030*
Leukocyte counts	1.366	1.119–1.666	0.002*
Treg cells	1.232	1.069–1.421	0.004*
CRP	2.036	1.035–4.005	0.039*
IL-10	1.290	1.065–1.563	0.009*
IFN-γ	0.913	0.842–0.994	0.040*

* Statistically significant.

CI = confidence interval; CRP = C-reactive protein; IFN = interferon; IL-10 = interleukin 10; OR = odds ratio; Treg = regulatory T.

### Correlation between lesion volume, NIHSS, and circulating inflammatory markers

Statistical evaluation revealed an independently positive correlation between lesion volume and CRP, IL-6, and IL-23 on day 3 after stroke onset (all p < 0.05). In addition, the serum level of IFN-γ was negatively associated with lesion volume on day 3 after stroke (p < 0.05; Table 5). However, the NIHSS at stroke onset showed an independent positive association only with IL-6 and IL-10 on day 3 (all p < 0.05; Table 5).

**Table 5:** Correlation coefficients between circulating inflammatory markers and lesion volume/NIHSS on day 3 after stroke in patients with infection

	TNF-α	CRP	IL-4	IL-6	IL-10	TGF-β	IL-17	IL-23	IFN-γ
LV									
*r*	0.201	0.340	0.020	0.296	0.030	0.025	0.109	0.312	-0.386
*p*	0.157	0.015*	0.891	0.035*	0.834	0.864	0.447	0.026*	0.005*
NIHSS									
*r*	0.181	0.271	0.206	0.289	0.835	0.093	0.191	0.030	-0.038
*p*	0.204	0.055	0.147	0.040*	<0.001*	0.516	0.180	0.833	0.793

* Statistically significant.

CRP = C-reactive protein; IFN = interferon; IL = interleukin; LV = lesion volume; NIHSS = National Institutes of Health Stroke Scale; TGF = transforming growth factor; TNF = tumor necrosis factor.

## DISCUSSION

In our study population, 53.1% of patients with ischemic stroke developed an infection, and the splenic volume of those patients decreased significantly in the early phase when compared with that of control subjects. Patients with ischemic stroke exhibited significant changes in the percentage of lymphocyte subpopulations and serum levels of inflammatory markers. The changes were similar in infected and uninfected patients when compared with control subjects, but changes in some lymphocyte subpopulations and certain inflammatory markers were more profound in infected patients than in uninfected patients. The splenic volume correlated positively with the percentages of B cells, Th cells, and cytotoxic T cells, but negatively with Treg cells after stroke. No significant difference in lymphocyte subpopulations was observed between patients with ACS and PCS after controlling for NIHSS. Infections were associated with splenic volume, leukocyte counts, the percentage of Treg cells, and serum levels of CRP, IL-10, and IFN-γ. Serum levels of CRP, IL-6, IL-10, and IL-23 were significantly correlated with lesion volume and/or NIHSS in patients with ischemic stroke. The results suggest that changes in the cellular immune system and circulating inflammatory markers may be associated with SIDS and clinical severity of the stroke.

Recent studies in rodents have shown that the spleen contracts in response to acute ischemic stroke [6, 20, 21]. In our clinical study, we observed only a slight contraction of the spleen in patients with ischemic stroke on day 3 after symptom onset and no significant difference in size from that of controls, consistent with findings from a previous study [22]. Further analysis of our results revealed that, after controlling for age, gender, and body mass index, the spleen of stroke patients with infection contracted significantly on day 3 after symptom onset when compared with that of control subjects. Our findings support the notion that a decrease in splenic volume may be associated with the occurrence of SIDS.

Previous reports are conflicting in regard to the effect of stroke on the frequency or percentage of Treg cells [7, 22–24]. Similar to our previous findings in an animal model [22], we found that the percentage of Treg cells in the lymphocyte population increased, whereas the percentages of Th cells, cytotoxic T cells, NK cells, and B lymphocytes all significantly decreased during the early phase after ischemic stroke. However, we only found significant difference in the percentage of Treg cells between patients with and without infection on day 3 after stroke onset. Because the inflammatory response is complex, we cannot exclude the possibility that a mixed consequence of infection itself may influence the cellular immune system and circulating inflammatory markers of stroke patients. Th cells aggravate inflammatory response, whereas Treg cells exert immunosuppressive effects in the process of inflammation [25, 26]. Some research has suggested that the imbalance in Th/Treg cells may contribute to immunosuppression [25]. Our results indicate that after ischemic stroke, the percentage of Treg cells increases while the percentages of Th cells, cytotoxic T cells, and NK cells decrease. These results support the notion that cellular immunity is depressed after ischemic stroke. We also evaluated the correlation between spleen size and lymphocyte subpopulations in this study. Previous research has indicated that SIDS may be caused by splenic cell death as well as an increased presence of CD4+FoxP3+ regulatory T cells [26]. Other research also showed that Treg cells are produced in the thymus as a mature cell population [26, 27]. Although our study illustrated that splenic volume correlated positively with the percentages of circulating B cells, Th cells, and cytotoxic T cells, but negatively with the percentage of circulating Treg cells after stroke, the mechanism that underlies changes in the cellular immune system and splenic volume needs additional investigation.

Cytokines such as IL-4, IL-10, and TGF-β have immunodepressive ability [9, 10, 28, 29]. In our study, the serum levels of TNF-α, CRP, IL-4, and IL-10 were all significantly increased in stroke patients, whereas, the serum level of IFN-γ was decreased compared with that in control subjects. The alterations in CRP and IFN-γ, were similar as above in stroke patients with and without infections. One prior study reported that a decrease in IFN-γ secretion contributed to spontaneous bacterial infections in stroke patients [30]. IFN-γ from Th cells is essential for host survival and enhances CD8 T cell function during infection [31, 32]. IL-10 and TGF-β are essential for the differentiation of Treg cells [13, 33]. Just as IL-10 and TGF-β contribute the differentiation of Treg cells, IL-17 and IL-23 contribute to the differentiation of Th cells [25]. Though we found an imbalance in Th/Treg cells, our research did not show a reduction in IL-17 or IL-23 in patients with ischemic stroke, a finding that may relate to the increase in frequency of total leukocytes in the early phase of stroke. These data indicate that changes in the circulating inflammatory markers complement alterations in the cellular immune system after ischemic stroke, which may contribute to or be a manifestation of post-stroke infection.

The incidence of pneumonia and urinary tract infection among stroke patients was 53.1% in our study but 21-65% in other studies [1, 2]. The incidence of infection in stroke patients was affected by NIHSS, lesion volume, age, gender, smoking, history of diabetes, and stroke subtype [34–37]. Patients with PCS always develop dysphasia, which is associated with pneumonia [3, 37]. The percentage of patients with PCS in our study was higher than that in a previous report [38]. More patients with PCS plus a higher smoking rate may have led to a higher infection rate in our study than in some previous reports [24, 39, 40]. The association between serum IL-10 level and infections in stroke patients has been reported [17, 41]. In this study, we further researched the relationship between lymphocyte subpopulations, cytokine levels, and infections after ischemic stroke. Adjusted logistic regression analysis of our data indicated that infections were not only associated with serum levels of IL-10, but also correlated with splenic volume, leukocyte counts, the percentage of Treg cells, and serum levels of CRP and IFN-γ.

Previous reports have shown that TNF-α, IFN-γ, IL-6, IL-17, IL-23, and some monocytes are detrimental in the pathologic process of stroke [42–46], whereas Treg cells, IL-4, IL-10, and TGF-β are major cerebroprotective modulators of post-ischemic inflammatory brain damage [47–49]. Studies also have shown that lesion volume correlates with IL-6 level after ischemic stroke [50]. Here, we showed that IFN-γ was negatively correlated with lesion volume on day 3 after stroke, but that CRP, IL-6, and IL-23 were positively correlated with lesion volume at that time point. However, only IL-6 and IL-10 were positively correlated with NIHSS on day 3 after stroke. These results indicate that immunologic changes were also associated with the degree of brain injury and the prognosis of patients with ischemic stroke. Although a crude association between admission CRP level and short-term functional outcome of stroke has been reported [51, 52], we did not identify an association between the serum CRP and NIHSS in our study, probably because of a relatively high incidence of infection in our patients.

SIDS occurs immediately after stroke in animals and patients [39, 53]. Some research has revealed that the nadir for frequency of CD3+ and CD4+ cells is on day 0 in stroke patients, suggesting that cellular immunodepression precedes infectious complications in humans [39]. Therefore, the first blood collection in this study might have been too late to show initial immunodepression. However, like other clinical studies of traumatic brain injury and stroke [17, 54], we showed that substantial decreases or increases in the percentage of lymphocyte subpopulations and serum levels of inflammatory markers are delayed compared with those shown in animal studies. Others have shown that aspirin, statins, and the inflammatory reaction itself may alter some of the inflammatory parameters [1]. Because most stroke patients in our study received aspirin and statins before the first blood draw, and infection occurs in the acute phase of stroke, we cannot determine whether the use of these drugs delayed changes in the systemic immune system or if the inflammatory reaction itself influenced the systemic immune system after stroke. Although our study revealed that the percentage of Treg cells and serum levels of CRP and INF-γ differ significantly between infected and uninfected patients on day 3, we cannot fully exclude the influence of hospital admission-associated anxiety and stress on the systemic immune system.

Our study is limited because it involved only one academic center. Validation in additional cohorts is needed to confirm our findings. Additionally, we assessed the splenic volume only on day 3 after stroke. Long-term outcome data were not available for this cohort. We did not observe significant differences in lymphocyte subpopulations between patients with ACS and PCS after controlling for NIHSS, possibly because of the small sample size. Despite these limitations, we showed the dynamic changes in the percentage of lymphocyte subpopulations and the relationship between lymphocyte subpopulations, cytokine levels, and infections. We also established a correlation between spleen size and lymphocyte subpopulations, and the effects of vascular territories on the severity of SIDS in the early phase of ischemic stroke. It would be interesting to analyze the predictive value of the initial levels of inflammation (which likely occur before infection). The knowledge gained will be useful for the treatment of ischemic stroke and its infectious complications.

## CONCLUSIONS

Ischemic stroke has a profound impact on the systemic immune system, and changes in the cellular immune system and circulating inflammatory markers may be associated with development of SIDS. Changes in the percentage of some lymphocyte subpopulations and in the serum levels of some inflammatory cytokines may reflect the degree of brain damage and the status of immunosuppression in ischemic stroke patients. Because infection may influence the recovery of stroke patients, regulating the imbalance in Th/Treg cells and associated cytokines may offer novel strategies for the treatment of stroke and related infections.

## MATERIALS AND METHODS

### Study population

Ninety-six patients with acute ischemic stroke were recruited into this study. Study entry criteria were the occurrence of an acute ischemic stroke within the previous 12 h, a score of at least 4 on the NIHSS at admission (because changes in the cellular immune system and circulating inflammatory markers were influenced by lesion volume in an animal model of hemorrhagic stroke [55]), and patient age of at least 18 years. Computed tomography (CT) and MRI scans were used for the diagnosis of all patients on admission. Exclusion criteria included clinical signs of infection on admission (e.g., pneumonia, urinary tract infection), abnormal laboratory test results (e.g., routine blood and urine, erythrocyte sedimentation rate, CRP, and calcitonin element), malignant tumor, participation in another interventional trial, immunologic disorders prior to stroke, usage of any medications (before or after stroke) known to interfere with cytokine signaling, intravenous or intra-arterial infusion of recombinant tissue plasminogen activator therapy during hospitalization, preceding or ongoing antibiotic therapy within the last 24 h, and immunosuppressant therapy within the last 30 days. Ninety-nine age-matched controls were also recruited. All of the control subjects came from the health checkup center of the Fifth Affiliated Hospital of Zhengzhou University, and their health status was known before physical examination and routine laboratory tests. The exclusion criteria were the same as those described above. Patient data and blood samples were handled in accordance with the published International Health Guidelines (Declaration of Helsinki, 2008). The study protocol was approved by the local ethics committee according to the World Medical Association as outlined in the Declaration of Helsinki. The families of all stroke patients and control subjects signed informed consent for aseptic peripheral venous puncture.

### Criteria for pneumonia or urinary tract infection

Infection was defined according to the previous PANTHERIS study [56]. Pneumonia was diagnosed by the presence of at least one of the A and one of the B criteria. Criteria A: abnormal respiratory examination, pulmonary infiltrates on chest x-ray; Criteria B: productive cough with purulent sputum, microbiological cultures from lower respiratory tract or blood cultures, leukocytosis, and elevation of CRP. For the diagnosis of urinary tract infection, two of the following criteria were included: fever ( > 38.0°C), urine sample positive for nitrite, leukocyturia, and significant bacteriuria. Infection also was diagnosed if temperature was above 38.0°C on at least two determinations and the patient had leukocytosis and positive blood cultures, but no determined focus.

### Measurement of spleen size

One certified sonographer measured spleen size using a GE Vivid E9 ultrasound machine and a phased sector array (5 to 1 MHz) transducer (GE Healthcare, New York, NY, USA) [21, 57]. The use of abdominal ultrasound for assessment of splenic volume has been validated previously against CT scan and autopsy measurements. The length, thickness, and width of the spleen at the splenic hilum level were measured in all patients on day 3 after stroke onset and in control subjects on the day after they signed the consent. Measurements were made in the sagittal (longitudinal) and transverse planes (measurement of width), and the maximum dimension was recorded in each plane. We calculated the final splenic volume as the average of three sets of these measurements (which have the least degree of variation among them) using the standard prolate ellipsoid formula, as has been reported previously [21]. The formula incorporated the product of one-dimensional diameters (W × T × L) into the equation V = (W × T × L ×π/6), where V is the ellipsoid volume, W is width, T is thickness, and L is length [21]. The measured volume was adjusted for patient height, weight, and gender as previously described [21].

### Flow cytometry

Peripheral blood samples (5 mL) were drawn from control subjects on the day after they signed the consent and from patients on days 1, 3, and 7 after stroke onset. Peripheral blood mononuclear cells were isolated within 2 h after blood draw. We used flow cytometry to determine the percentage of each lymphocyte subset within peripheral blood lymphocytes: CD45 for lymphocytes; CD3 and CD4 for Th cells; CD3 and CD8 for cytotoxic T cells; CD56 and CD16 for NK cells; CD19 for B lymphocytes; CD4, CD25, and FoxP3 for Treg cells (all antibodies from BD Pharmingen, San Diego, CA). Cells were phenotyped by four-color flow cytometry on a FACSCalibur flow cytometer using CELLQuest software (BD Biosciences, San Jose, CA, USA). Lymphocytes were gated based on a previous report [58]. Dead cells were excluded if aqua amine-reactive dye was positive. Treg cells were gated within the CD4+ cells. Other lymphocyte subsets were gated within the viable lymphocyte gate [58]. Based on the results of a routine blood test, the number of leukocytes was also recorded on admission.

### ELISA

Using blood samples collected from patients and control subjects as described in the flow cytometry section, we measured protein concentration with the bicinchoninic acid assay kit (BCA; Pierce, Appleton, WI, USA). The total protein concentration was adjusted to 1 mg/mL. The concentrations of TNF-α, CRP, IL-4, IL-6, IL-10, IL-17 (Sigma-Aldrich, St. Louis, MO, USA), IFN-γ (eBioscience, San Diego, CA), TGF-β (active; R&D Systems, Minneapolis, MN), and IL-23 (BioLegend, San Diego, CA, USA) were quantified by ELISA kits. Levels of TNF-α, CRP, IL-4, IL-6, IL-10, and IL-17 were measured within the range of the standard curve. The measurement range for the IFN-γ kit was 0.016 to 10 pg/mL, that for the TGF-β kit was 31.2 to 2000 pg/mL, and that for the IL-23 kit was 31.3 to 2000 pg/mL.

### Assessment of lesion volume in patients with ischemic stroke

All MRI scans were acquired on a 3.0-Tesla system (Philips Medical System, Bothell, WA, USA) according to a previously described imaging protocol within 2 days after stroke onset [59]. The lesion volume was assessed as previously reported in patients with ischemic stroke [60]. We saved all diffusion-weighted imaging (DWI) scans in Digital Imaging and Communications in Medicine format for importation into ImageJ software using Sync Measure 3D plugin software (National Institutes of Health, Bethesda, MD). We used these data to calculate infarct areas extracted for evaluation. Areas of infarction on DWI were highlighted by interactively setting a threshold of 3000 over the signal value. Then the threshold image was analyzed by using the particles command in Image J. The voxel size of each image was determined and used to calculate the infarction volume (1 voxel represents 1.875 × 1.875 × 5.0 mm).

### Data presentation and statistical analysis

Statistical analysis was carried out with SPSS 13.0 (SPSS Inc., Chicago, IL, USA). Data are presented as mean (SD) or median (range) for continuous variables and percentage (count) for categorical variables. Chi-squared or Fisher's exact test was used for analysis of categorical data, as appropriate. Demographic variables and comorbidities were compared between stroke patients and control subjects with Student's t test. Spleen size was compared among stroke patients, uninfected and infected patients, and control subjects with one-way ANOVA followed by Bonferroni correction. The body temperature, median frequency of leukocytes, lymphocyte subpopulations, and serum level of circulating inflammatory markers were compared among stroke patients and control subjects at each time point with one-way ANOVA followed by Bonferroni correction. The lymphocyte subpopulations and serum levels of circulating inflammatory markers were also compared between infected and uninfected patients at the three time points with repeated measures ANOVA followed by Bonferroni correction. Analysis of covariance was used to compare the difference in lymphocyte subpopulations between patients with PCS or ACS after controlling for the NIHSS at the three time points. All continuous variables with the characteristic of homogeneity of variance distributed normally. The Tukey-Kramer adjustment was used to balance the sample size in different groups. Because of the small sample size, we did not compare differences in lymphocyte subpopulations or serum level of circulating inflammatory markers for each infection type (pulmonary infection, sepsis, and urinary tract infection). We used logistic regression to determine the relationship between cytokine levels, lymphocyte subpopulations, and infections. Pearson product-moment correlation was used for analysis of correlation between circulating inflammatory markers and lesion volume or NIHSS. The correlation between splenic volume and lymphocyte subpopulations was also evaluated with analysis of Pearson product-moment correlation. Statistical significance was set at p < 0.05.
